# Solvent-Assisted Ketone Reduction by a Homogeneous Mn Catalyst

**DOI:** 10.1021/acs.organomet.2c00077

**Published:** 2022-04-15

**Authors:** Annika M. Krieger, Vivek Sinha, Guanna Li, Evgeny A. Pidko

**Affiliations:** † Inorganic Systems Engineering Group, Department of Chemical Engineering, Delft University of Technology, Van der Maasweg 9, 2629 HZ Delft, The Netherlands; ‡ Biobased Chemistry and Technology, Wageningen University, Bornse Weilanden 9, 6708WG Wageningen, The Netherlands; § Laboratory of Organic Chemistry, Wageningen University, Stippeneng 4, 6708WE Wageningen, The Netherlands

## Abstract

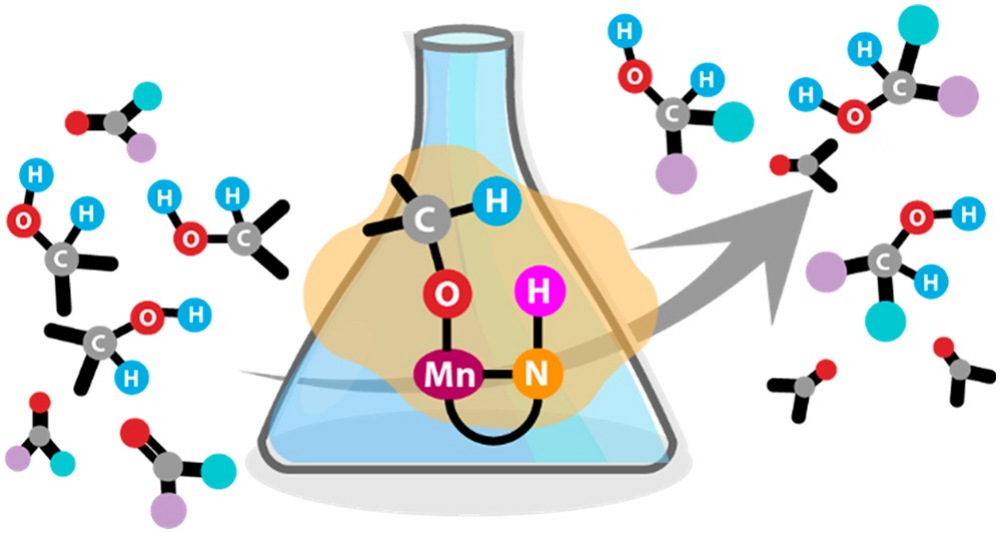

The choice of a solvent and the reaction conditions often defines the overall behavior of a homogeneous catalytic system by affecting the preferred reaction mechanism and thus the activity and selectivity of the catalytic process. Here, we explore the role of solvation in the mechanism of ketone reduction using a model representative of a bifunctional Mn-diamine catalyst through density functional theory calculations in a microsolvated environment by considering explicit solvent and fully solvated ab initio molecular dynamics simulations for the key elementary steps. Our computational analysis reveals the possibility of a Meerwein–Ponndorf–Verley (MPV) type mechanism in this system, which does not involve the participation of the N–H moiety and the formation of a transition-metal hydride species in ketone conversion. This path was not previously considered for Mn-based metal–ligand cooperative transfer hydrogenation homogeneous catalysis. The MPV mechanism is strongly facilitated by the solvent molecules present in the reaction environment and can potentially contribute to the catalytic performance of other related catalyst systems. Calculations indicate that, despite proceeding effectively in the second coordination sphere of the transition-metal center, the MPV reaction path retains the enantioselectivity preference induced by the presence of the small chiral *N*,*N*′-dimethyl-1,2-cyclohexanediamine ligand within the catalytic Mn(I) complex.

## 1 Introduction

Transfer hydrogenation (TH) is a simple and robust chemical transformation that can be used widely in both achiral and chiral synthesis.^1^ During the reaction, a hydrogen donor molecule, present in excess, donates an equivalent of H_2_ to reduce a polar moiety in a substrate, e.g., to transform a carbonyl group to a hydroxyl group. The reaction effectively shuffles dihydrogen from one alcohol to another. To control the selectivity of these reactions and the efficiency of the catalyst, it is imperative to understand the underlying mechanism in great detail. Besides understanding and predicting the reactivity of a catalyst, the knowledge of a mechanism allows chemists to make great steps toward the optimization of the chemical transformation. On one hand, if the mechanism of a process is known, deactivation pathways can be analyzed and strategies can be implemented to prevent the catalyst from escaping the productive catalytic cycle.^2^ On the other hand, the reactivity, substrate scope, and efficiency of a process can be tuned by fine-tuning and optimizing the molecular design of the catalyst.^3,4^

**Scheme 1:**
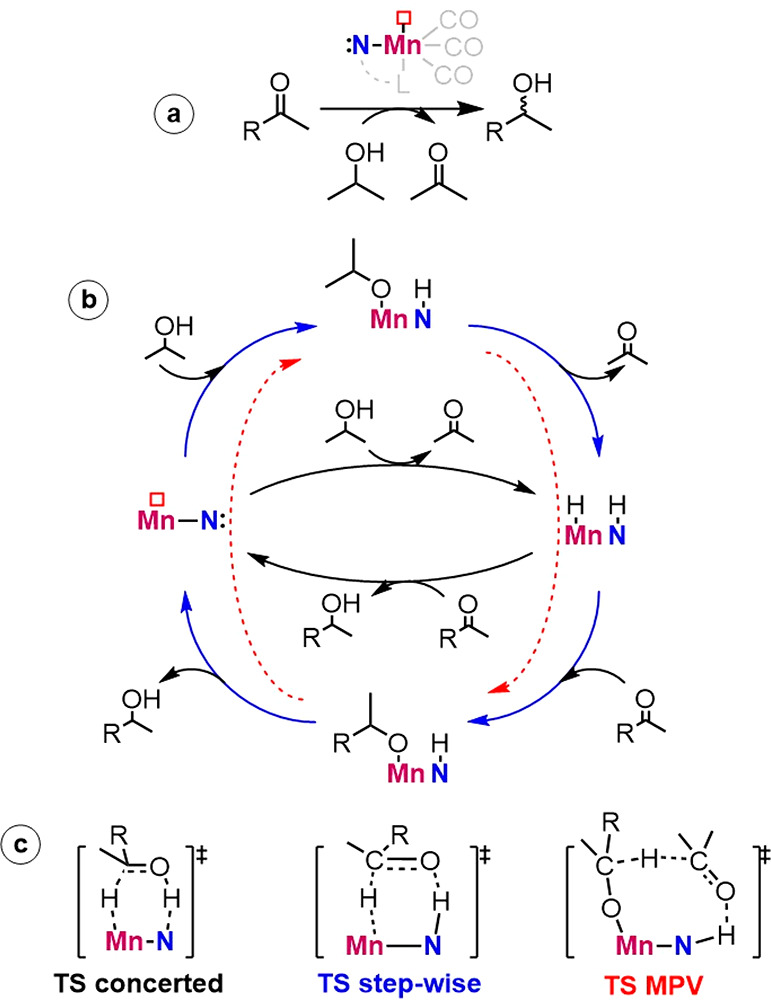
(a) Transfer hydrogenation of ketones by a Mn–diamine model catalyst. (b) The catalytic cycle depicts the three different routes for the TH reaction over the Mn–N active site, namely the concerted (black) and step-wise (blue) metal–ligand cooperative mechanism and the outer-sphere MPV reduction path (red). (c) Schematic illustration of the key hydride transfer transition states for the respective paths.

The TH reaction can be viewed as a combination of two events, namely the oxidation of the alcohol and the reduction of an unsaturated group, e.g., a C=O moiety, in the substrate. In the first oxidation step, the alcohol is dehydrogenated over the metal–ligand acid–base pair (M–L = Mn–N in Scheme 1) to produce the M(H^–^)–-L(H^+^) complex. In the next reduction step, a substrate with a C=O function accepts the hydride from M(H) and a proton from L(H) to complete the TH event. Two primary mechanisms have been discussed in the literature for the TH reaction by bifunctional transition-metal catalysts, namely the concerted and stepwise mechanisms (Scheme 1).^5−11^ In the concerted mechanism, the hydride and proton transfer steps occur within the same transition state (TS), which typically features a six-membered pericyclic configuration. In contrast, in the stepwise mechanism, the L–H moiety remains protonated during the hydride transfer TS, and the protonation of the alkoxide moiety at the final stage of the catalytic cycle proceeds in a separate elementary step.

The key assumption in both mechanisms is that the ligand directly participates in the catalytic process.^12^ In the concerted mechanism, the ligand functions as reversible storage for H^+^. In the stepwise mechanism, the L–H moiety favorably orients the alkoxide moiety, which transfers the hydride, and polarizes the C=O function for favorable hydride insertion during the reduction event. The ability to reversibly protonate and deprotonate the nitrogen center on the ligand is dictated by the p*K*_a_ value, which has been proposed to be a determining factor between the concerted mechanism and the stepwise mechanism.^13−17^

Interestingly, the Meerwein–Ponndorf–Verley (MPV) mechanism, often considered for reduction reactions by metal alkoxides, has not been discussed in the literature for TH reactions catalyzed by transition-metal complexes.^18−21^ In the MPV mechanism, the proton and hydride transfers occur directly between the reducing agent and the substrate via a cyclic TS facilitated by coordination to the Lewis acidic metal center. The MPV mechanism excludes the formation of a metal hydride species, and the ligand is not expected to play a direct role. Earlier, a MPV-like mechanism for the catalytic reduction of ketones was eliminated from mechanistic considerations because of the unfavorable energetics computed for the respective cyclic transition state.^5,7^

Improvements in computational strategies enable us to incorporate solvation effects more efficiently^15,17,22,23^ and reconsider mechanisms that were previously discarded.^24−26^ Therefore, we herein reconsider the feasibility of an MPV-like mechanism for asymmetric ketone reduction by a bifunctional Mn–diamine catalyst when explicit solvent description is applied. The *cis*-Mn(*N*,*N*′-dimethyl-1,2-cyclohexanediamine)(CO)_3_Br catalyst was chosen for this study due to its generic features, which make it an attractive representative model system for mechanistic studies.^27^

## 2 Results and Discussion

**Figure 1:**
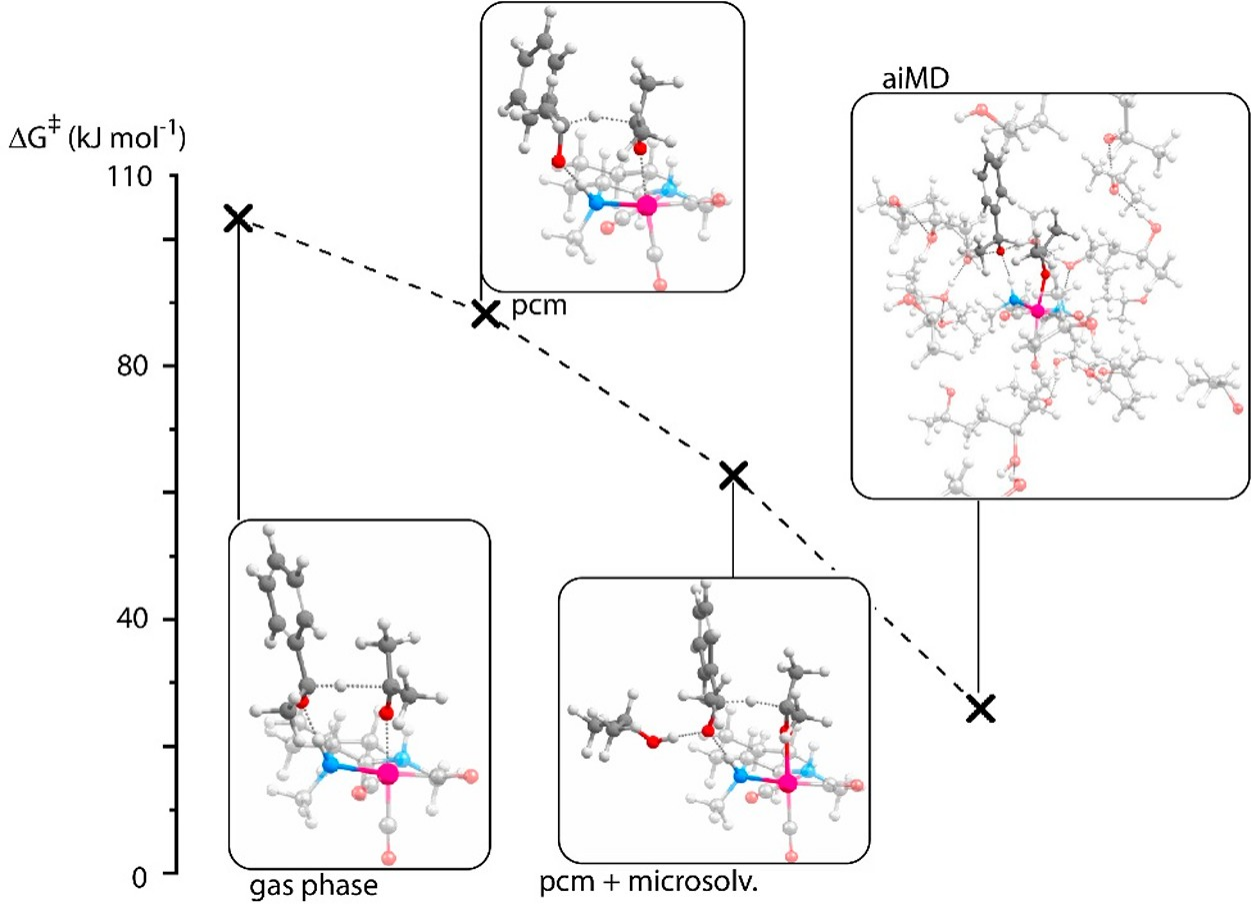
Gibbs free-energy barriers and transition-state structures of the hydride transfer for **I** → **II** in the gas-phase model, the implicit PCM solvation model, a microsolvated model incorporating PCM solvation and one additional solvent molecule, and a fully explicit solvation AIMD model. Energies are in kilojoules per mole. The free-energy barriers for the molecular models were computed using the hybrid PBE0 exchange-correlation functional, whereas the periodic full explicit solvation AIMD model was treated at the GGA (BLYP) level of theory, which may have underestimated the barrier heights.

Calculations to capture the effect of the solvent environments have been carried out with gas-phase and microsolvated molecular models using density functional theory (DFT) calculations at the PBE0-D3(CPCM)/6-311+G(d,p) level of theory in addition to fully explicit solvated ab initio molecular dynamics (AIMD) simulations. Our results reveal the active participation of the solvent in the transfer hydrogenation of the ketone by the Mn catalyst. Analysis of solvated intermediates via AIMD revealed that, in addition to the substrate isopropanol, an additional isopropanol moiety from the solvent plays an important role in the mechanism. On the basis of the AIMD results, we constructed a microsolvated model on which DFT calculations were performed for the detailed and accurate analysis of the minimum-energy reaction pathway (MERP). The comparison of the barriers for the key H-transfer step obtained with these different models is presented in Figure 1. The results of the DFT calculations and the AIMD analysis will be discussed consecutively.

### 2.1 DFT Calculations

**Scheme 2:**
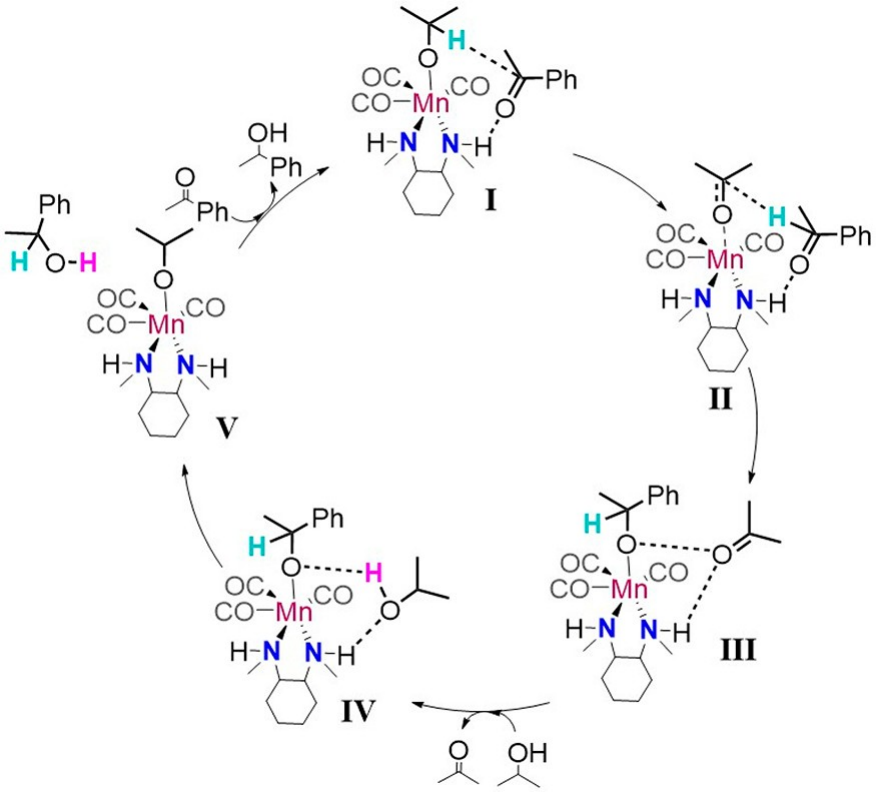
Proposed Meerwein–Ponndorf–Verley-Type Mechanism for Ketone Transfer Hydrogenation by the Mn-NN Catalyst Facilitated by Isopropanol Solvent Molecules

**Figure 2:**
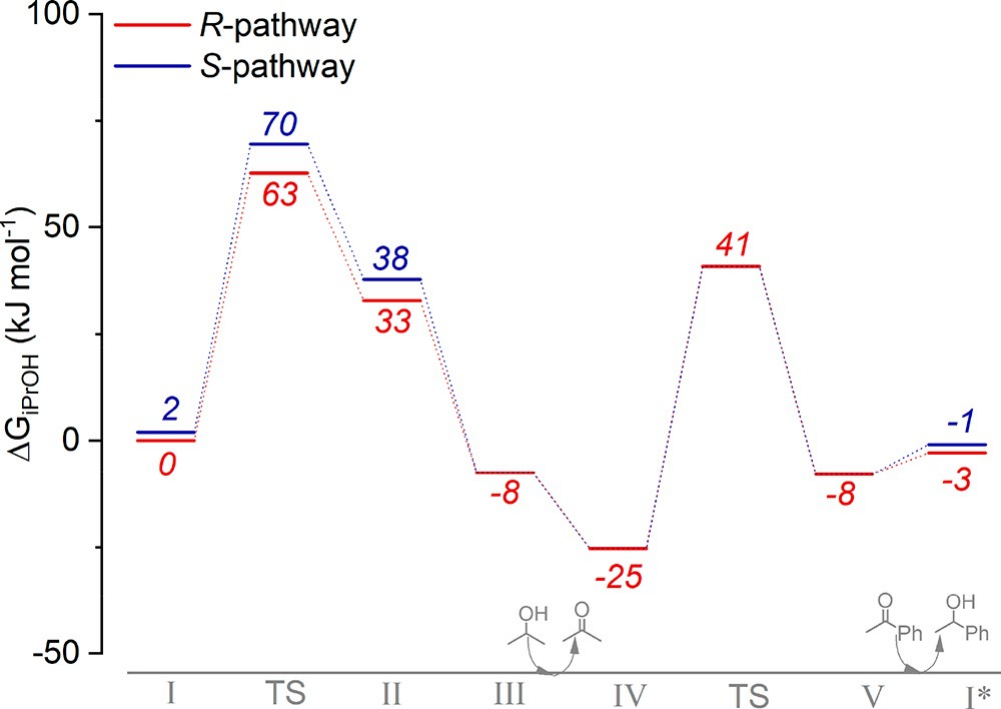
Reaction Gibbs free-energy diagram for the reduction of acetophenone to (red) (*R*)- and (blue) (*S*)-1-phenylethanol in the microsolvation model.

The microsolvated model included two isopropanol solvent molecules, one acting as the reactant hydrogen donor and one merely participating by stabilizing the intermediate and transition state structures. In addition, continuum solvation in isopropanol was applied using the CPCM solvation model.^28^ On the basis of the calculations, a new catalytic pathway is proposed as schematically shown in Scheme 2, with the reaction free energy diagram shown in Figure 2.

**Figure 3:**
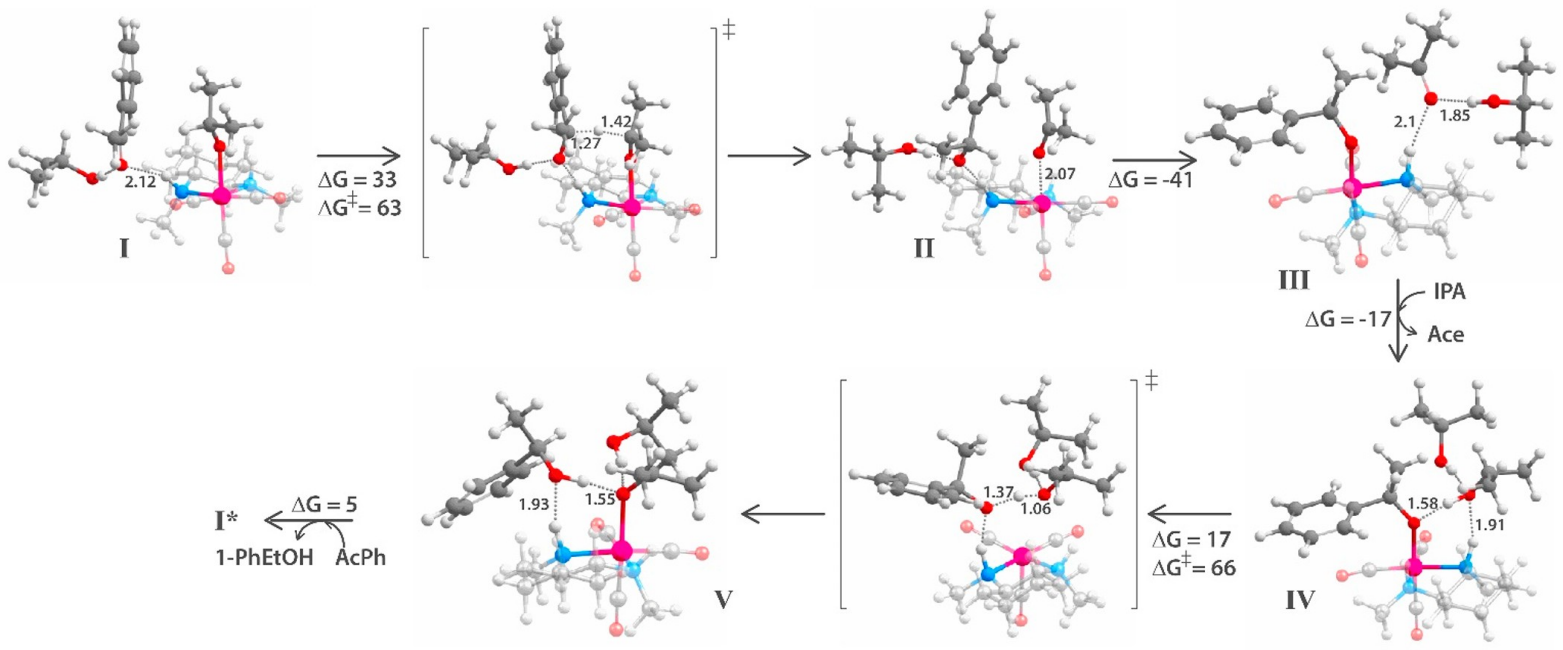
Structures of the reaction intermediates and transition states of the MERP for the reduction of acetophenone to (*R*)-phenylethanol. Gibbs free-energies are in kilojoules per mole, and bond distances in angstroms.

The reaction starts with the state **I**, in which the Mn–alkoxide (isopropoxide) complex forms a hydrogen-bonded complex with the solvated ketone substrate (acetophenone) at the NH moiety of the ligand. The hydride is transferred in an endergonic process (Δ*G* = 33–35 kJ mol^–1^ for the *R* and *S*-paths) to the acetophenone with a barrier of 63–68 kJ mol^–1^, resulting in an acetone molecule coordinated to the Mn center and a PhEtO^–^ anionic species in the second coordination sphere. This free alkoxide is stabilized by the hydrogen bonds with the second NH moiety and the additional isopropanol molecule (structure **II**, Figure 3). The weakly bound acetone is then readily replaced with the alkoxide to form the 1-phenylethoxide–Mn complex **III**. Next, an isopropanol molecule hydrogen-bonded with the NH moiety (**IV**) transfers its proton to the PhEtO^–^ species to form the phenylethanol product, which in turn forms a hydrogen bond with the NH moiety of the ligand. This step proceeds with a free-energy barrier of 66 kJ mol^–1^. Simultaneously, the formed iPrO^–^ binds with the undercoordianted Mn center to form **V** and thus closes the catalytic cycle.

The amino moiety on the ligand does not participate directly in the key elementary steps, with the exception of providing a precoordination handle that facilitates the reactive arrangement of the substrates in the vicinity of the Lewis acidic Mn site. A similar solvent-assisted proton-transfer function of the cooperative ligand sites was proposed for Noyori-type Ru hydrogenation catalysts by Dub et al.^15^ Our calculations highlight the importance of solvent participation in the catalytic carbonyl reduction by cooperative Mn(I) complexes. Most of the prior mechanistic proposals on the stepwise and concerted pathways for such systems imply that the N–H functionality is deprotonated and then protonated to transfer the hydrogen equivalent. In the current mechanism, the hydrogen transfer occurs directly from the solvent to the substrate. During the simulation, it was verified that the N–H moiety stays protonated throughout the reaction pathway. A similar effect was observed before for the (de)protonation steps in the reduction paths over Ru–H catalytic species.^23,26^ We estimated the p*K*_a_ values for both N–H sites using DFT calculations. The estimated value of p*K*_a_ ∼ 40 (in tetrahydrofuran) is consistent with our mechanistic finding that the N–H moiety remains protonated throughout the computed MERP.

While the NH functionality of the ligand backbone is not directly involved and stays protonated during the hydride transfer, it plays a crucial role in aligning the substrates to enable the hydride transfer. The stabilizing influence of the solvent environment for the rate-determining hydride transfer step (**I** → **II**) becomes apparent when the energetics are computed with different models, namely when the gas-phase, implicit solvation, and explicit (micro)solvation models are compared (Figure 1). The Gibbs free energy of activation for the transformation of **I** to **II** is the highest in the gas phase (103 kJ mol^–1^), followed by the implicit solvation model (83 kJ mol^–1^) and the microsolvation model (63 kJ mol^–1^). Thus, the introduction of the implicit solvation stabilizes the TS by 20 kJ mol^–1^. The inclusion of microsolvation via an additional isopropanol molecule further reduced the barrier by another 20 kJ mol^–1^. Our previous computational studies showed that the alternative one-step ketone reduction by a Mn–H species (Scheme 1) proceed with the barriers in the range 70–80 kJ mol^–1^.^27,29^ These results suggest that the current solvent-assisted MPV-type outer-sphere reduction by Mn–alkoxide complexes can represent a viable competitive mechanism.

The complete reaction path for this mechanism was computed for both *R* and *S*-enantiomers, and the resulting reaction Gibbs free energy diagram is shown in Figure 2. The first transition-state barrier induces the chirality of the final acetophenol product. Therefore, the pathway from intermediate **III** to intermidate **V** has only been investigated for the *R*-geometry. The optimized geometries of all intermediates and transition states toward the formation of (*R*)-1-phenylethanol are presented in Figure 3. As highlighted above, the solvent environment plays a crucial role in the reaction mechanism. The stabilization of the anionic intermediates via hydrogen bonding interactions with the solvent significantly reduces the reaction barriers, as illustrated in Figure 1. The energetics of the catalytic reaction critically depend on the configuration of the microsolvation environment within the molecular model. We have considered an alternative reaction path with different coordination for the second isopropanol molecule. An alternative configuration of species **I** in which the explicit solvation is provided to the Mn–isopropoxide moiety instead of the acetophenone substrate results in a much higher free-energy barrier for the hydride transfer reaction of over 100 kJ mol^–1^ and is an overall less favorable catalytic path (see S2 in the Supporting Information, Figures S3.1–3.3).

The more facile reduction of acetophenone to (*R*)-penylethanol compared to its stereoisomer indicates that the asymmetric ligand backbone is able to induce chirality in the MPV mechanism. Our calculations show that within the constraints of the microsolvation model the enantioselectivity-determining transition state (**TS**_**I–II**_) for the hydride transfer has a barrier comparable to that for the subsequent proton-transfer step. The cooperation between the NH moiety of the ligand and the reactive Mn Lewis acidic site facilitates the reaction. The NH site helps direct the proton- and hydride-donor molecules to facilitate their reactions along the catalytic mechanism. The difference between the formation of the *R*- and *S*-enantiomers is 7 kJ mol^–1^, which corresponds to an enantiomeric excess of 85% and is in line with the trend observed in experimental studies.^27^

### 2.2 AIMD Simulation

**Figure 4:**
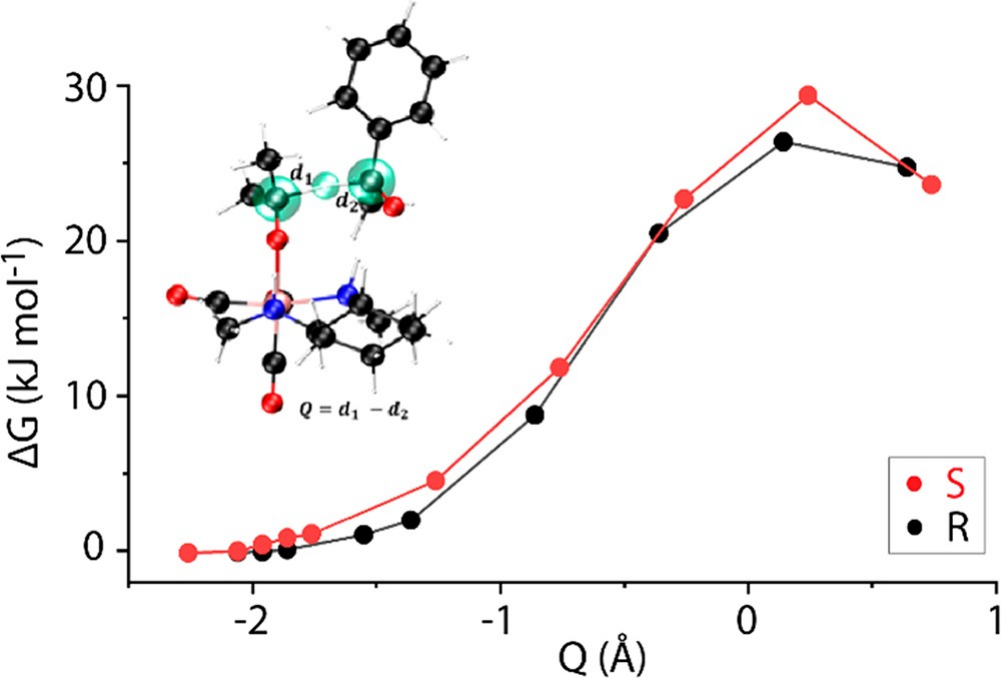
Gibbs free-energy profiles for transfer of hydride from the iPrO adducts to a solvated acetophenone moiety along the *S*- and *R*-pathways. A depiction of the reaction coordinate (*Q*) for hydride transfer in the AIMD simulations is displayed in the left corner. The C and H atoms directly involved in *Q* are highlighted in green.

The microsolvation DFT results presented above reveal the critical role the relative arrangement of the first solvent shell plays in the reaction path. To eliminate the potential artifacts due to the arbitrary configuration selection and to further investigate the explicit role of the solvent in the hydrogenation reaction, we carried out ab initio molecular dynamics (AIMD) simulations with a full periodic explicit solvation shell on the key hydride transfer step for the formation of both *R* and *S*-enantiomers. We considered a one-step hydride transfer pathway from an iPrO adduct to an acetophenone moiety in the solvent phase. The difference in the C–H bond lengths of the donor and acceptor C moieties was considered as the reaction coordinate *Q* (Figure 4). *Q* < 0 corresponded to a “reactant-like” state of the system, while *Q* > 0 corresponded to a “product-like” state.

**Figure 5:**
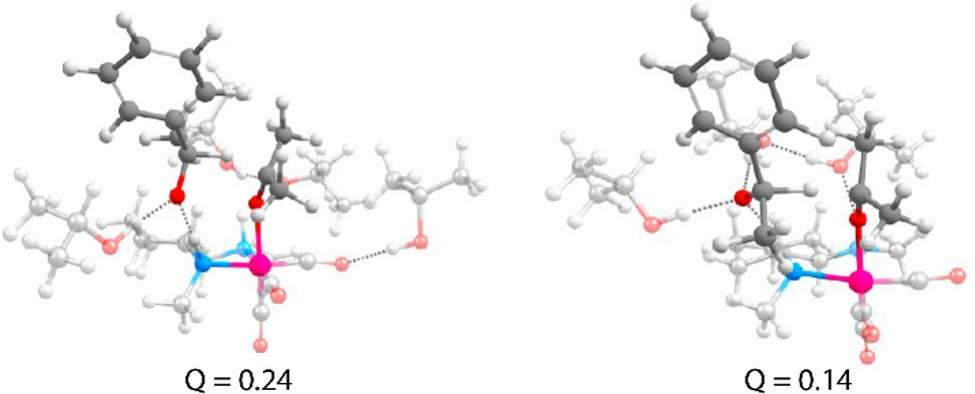
Snapshots of solvated geometries close to the transition state for the (left) *S*- and (right) *R*-isomers. The AIMD model inspired the placement of the solvent for DFT calculations.

Constrained AIMD simulations were performed for several fixed values of *Q* (10 values of −2.26 Å ≤ *Q* ≤ 0.64 Å for *R* and 10 values of −2.36 Å ≤ *Q* ≤ 0.74 Å for *S*). The resulting Gibbs free-energy profiles are shown in Figure 5. The hydride transfer was found to proceed with a free-energy barrier of 29 kJ mol^–1^ for the *S*-pathway and that of 26 kJ mol^–1^ for the *R*-pathway. Consistent with DFT-computed MERP, the *R*-pathway was found to proceed with a slightly lower barrier.

Analysis of geometries close to the TS revealed that steric hindrance in the *S*-isomer leads to less H-bonding stabilization of the O moiety in acetophenone than that in the *R*-isomer (Figure 5). This leads to a lower barrier for the *R*-path. Furthermore, an analysis of AIMD-computed trajectories showed that the N–H moiety remained protonated during the hydride transfer reaction, which is consistent with the DFT-computed mechanism.

Thus, for the current Mn–diamine catalytic system, our calculations show that the alkoxide adduct can represent the catalytically active species in ketone transfer hydrogenation. Such alkoxide complexes are commonly considered resting states in the alternative direct H_2_ hydrogenation reactions for which the involvement of the Mn–hydride active species is necessary.^27,30^ This provides a rationale for the divergent reactivity of Mn–diamine and related catalysts in the TH and direct H_2_ hydrogenation reactions.

## 3 Conclusion

In this work, by computationally considering a representative example of a Mn(I)–NN bidentate catalyst, we have shown that a MPV-type mechanism can be feasible for the transfer hydrogenation of ketones by a bifunctional homogeneous transition metal catalyst. Contrary to the conventionally proposed Mn-mediated hydride transfer mechanisms, the reduction of the ketone substrate by the Mn–alkoxide adduct proceeds via a solvent-assisted direct hydride shift. The Mn center acts as a Lewis acid, polarizing the substrate and stabilizing the anionic intermediates. The N–H functionality of the bifunctional catalyst remains intact in the course of the reaction while it facilitates the reduction reaction by directing the coordination of the substrate. Importantly, the ligand is not deprotonated during the catalytic reaction and therefore is not directly involved in the reduction. Our DFT and AIMD calculations have shown that the N–H moiety serves as a supramolecular directing group by ensuring the reactants are positioned in a proper orientation by H-bonding. The ketone is instead directly hydrogenated by the isopropanol solvent. The role of the solvent description is crucial, as including solvation not only significantly lowers the transition-state barrier for such a path but actually enables the identification of the respective reaction channel. This work shows that the Mn–alkoxide species can be the active species for ketone transfer hydrogenation. The retention of the N–H bond is also possible in reductions where small molecules act as hydrogen donors. The inclusion of additional solvent molecules in the theoretical description of the catalytic system is crucial for the feasibility of the mechanism.

## 4 Computational Details

All DFT calculations were performed using the Gaussian 16 rev. C0.1 program.^31^ The hybrid exchange-correlation functional PBE0^32^ was used in combination with the 6-311+G(d,p) basis set on all atoms for both geometry optimization and vibrational analysis. van der Waals interactions are accounted for by the dispersion-corrected DFT-D3 (BJ) method.^33^ The ultrafine grid was used uniformly. The nature of each stationary point was confirmed by frequency analysis, confirming there were zero imaginary frequencies for minima and one for the transition states. Gibbs free energies (Δ*G*) were calculated at a temperature of 333.15 K with a CPCM solvent correction for isopropanol.^34^

The DFT-based Born–Oppenheimer molecular dynamics simulations were performed with the CP2K package^35^ and using the BLYP functional^36,37^ supplemented by D3 dispersion corrections.^38^ The system consisted of complexes I-R and I-S with 22 isopropanol molecules in a periodic cubic box (*L* = 15 Å). The initial configuration of the solvent box containing the Mn complex was created by adding iPrOH solvent molecules to DFT-optimized geometries of Mn complexes using the GROMACS suite of software. AIMD simulations of the solvated complexes were performed in the NVT ensemble for a long simulation time (>20 ps). A time step of 1.0 fs was used in our simulations. NVE simulations with a 1 fs time-step confirmed that there was no appreciable drift in the total energy of the system. The temperature was controlled by a CSVR thermostat^39^ and was set at *T* = 360 K. Goedecker–Teter–Hutter (GTH) pseudopotentials were employed to account for the interactions of the nuclei and core electrons with the valence electrons. The electronic states were expanded using a DZV-GTH-PADE basis set for manganese and a TZVP-GTH basis set for all other atom types. The auxiliary plane waves were expanded up to 280 Ry.^13,26^ We used the constrained molecular dynamics method^37,40^ to determine the Gibbs free energy profile. Using a chosen reaction coordinate, *Q*, simulations were performed at several fixed values of *Q*. The change in free energy upon changing from *Q*_1_ to *Q*_2_ was then computed according to eq 1. Here, ⟨*F*(*Q*)⟩ is the average constraint force measured for each value of *Q*. For each value of the reaction coordinate, 15–20 ps runs were performed. The average force was computed over the last 5 ps of the production run, and the standard deviation of the computed average force was found to be negligible for all values of *Q*.
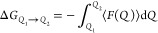
1

## Supporting Information Available

Optimized structures of the reaction intermediates and transition states, apparent activation energies, energy terms from the DFT calculation, and alternative pathway (PDF)
Optimized structures (XYZ)

## Supplementary Material

om2c00077_si_001.pdf

om2c00077_si_004.xyz
