# Supplementary material for: Solvent-Assisted Ketone Reduction by a Homogeneous Mn Catalyst
Source: Organometallics. 2022 Apr 15;41(14):1829–35. doi: 10.1021/acs.organomet.2c00077 (PMC9326964; doi:10.1021/acs.organomet.2c00077)
Supplement: Supplementary file 1 — om2c00077_si_001.pdf [file om2c00077_si_001.pdf]

SUPPORTING INFORMATION

Solvent-Assisted Ketone Reduction by a Homogeneous Mn-Catalyst

Annika M. Krieger[a], Vivek Sinha[a], Guanna Li[b, c], Evgeny A. Pidko[a]

[a] *Inorganic Systems Engineering Group, Department of Chemical Engineering, Delft University of Technology, Van der Maasweg 9, 2629 HZ Delft, The Netherlands*

[b]*Biobased Chemistry and Technology, Wageningen University, Bornse Weilanden 9 6708WG Wageningen, The Netherlands*

[c]*Laboratory of Organic Chemistry, Wageningen University, Stippeneng 4, 6708WE Wageningen, The Netherlands*

Corresponding author: Evgeny A. Pidko (E.A.Pidko@tudelft.nl)

Table of Contents

S1. Energetics of optimized structures....3
S2. Solvent arrangement....3
S3. Alternative pathway with smd model....4
S4. Energetics of optimized structures of the alternative pathway....6

S1. Energetics of optimized structures

Free molecules

Species	E	ZPE	E_{therm}	S
**Acetone**	-192.989169	-192.905543	56.443	74.339
**Isopropanol**	-194.189087	-194.081625	71.228	71.906
**1-Phenylethanol**	-385.754344	-385.592602	107.944	93.996
**Acetophenone**	-384.555485	-384.417201	92.784	91.243

Reaction Intermediates

Species	E	ZPE	E_{therm}	S
**I R**	-2687.758353	-2687.117571	433.026	266.652
**I S**	-2687.756568	-2687.115777	433.055	268.679
**II R**	-2687.747121	-2687.107984	431.570	259.830
**II S**	-2687.744030	-2687.104914	431.665	262.454
**III R**	-2687.759750	-2687.119357	432.936	269.170
**IV R**	-2688.972501	-2688.306440	448.413	257.331
**V R**	-2688.971601	-2688.302914	449.715	250.502
**II R gas phase**	-2493.538263	-2493.006823	359.177	230.498
**II R pcm**	-2493.551149	-2493.020179	358.774	225.985
**IV alternative**	-2688.979466	-2688.313753	448.332	259.446

Transition States

Species	E	ZPE	E_{therm}	S
**$TS_{I-II\ R}$**	-2687.735325	-2687.097879	429.942	255.746
**$TS_{I-II\ S}$**	-2687.734639	-2687.096814	430.011	252.375
**TS_{IV-V}**	-2688.942395	-2688.280224	445.864	259.000
**$TS_{I-II\ R\ gas}$**	-2493.502000	-2492.973526	356.325	216.141
**$TS_{I-II\ R\ pcm}$**	-2493.518853	-2492.990680	356.027	215.289

E: Electronic energy (PBE0-D3/6-311+G(d,p)// PCM) [Hartree]
ZPE: Zero-point Energy [Hartree]
E_{therm}: Internal Thermal Energy [Kcal/Mol] at 333K
S: Entropy from translational degrees of freedom [Cal/Mol-Kelvin] at 333K

S2. Solvent arrangement

Alternative solvent arrangement in DFT calculations can drastically change calculated energetics. Therefore, our DFT calculations were complimented with an AIMD model to confirm the observed reaction pathway. In the following figure, we highlight the effect of solvent arrangement and show how small changes influences energetics. A rotation of 90 degree of the isopropanol molecules lead to an energy change of more than 20 kJ mol^{-1}. During the rotation, no bonds are broken, simply a change of solvent arrangement takes place.

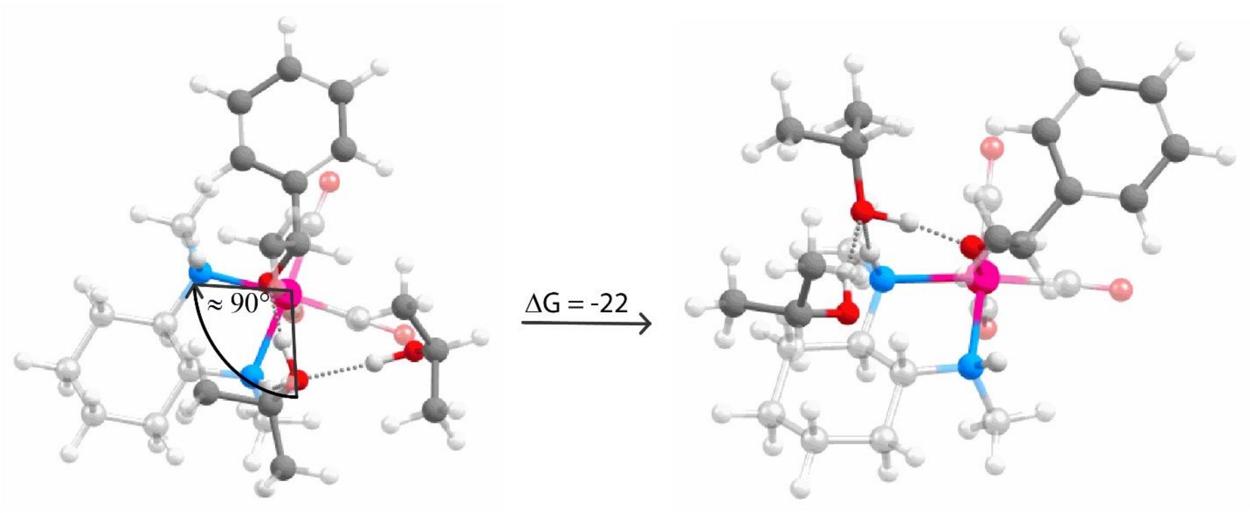

Figure S1: Two solvent arrangements for structure **IV** and the respecitive energy difference in kJ mol^{-1}.

S3. Alternative pathway with smd model

This pathway has been calculated as an alternative for the one presented in the article with an smd solvent model. In the alternative pathway, the reaction starts with the active species **I**, in which the Mn-alkoxide (isopropoxide) complex forms a hydrogen bonded complex with the ketone substrate (acetophenone) at the N_1H-moiety of the ligand. Next, the alkoxide anion is replaced with the acetophenone from the Mn center to form the activated species **II.** This steps proceeds endergonycally (ΔG = 74 kJ mol^{-1}) due to the energy loss associated with the formation of a separate ion pair between the isopropoxide anion and the cationic Mn complex. The isopropoxide ion is stabilized by hydrogen bonds with the N_2H-moeity and the additional isopropanol molecule (Structure II, Figure 4). At the next step, the hydride is transferred from the *i*PrO⁻ ion to the Mn-bound acetophenone. This reaction is strongly exergonic (ΔG = -68 kJ mol^{-1}) and proceeds with a barrier of only 35 kJ mol^{-1}. The hydride transfer yields acetone byproduct and the alkoxide (1-phenylethoxide) complex **III**. An isopropanol molecule hydrogen-bonded with the N_1H-moeity (**IV**) next transfers its proton to the alkoxide species to form phenylethanol, which, in turn, hydrogen-bonds with the N_2H-moeity of the ligand. Simultaneously, the formed iPrO⁻ binds with the undercoordianted Mn center to form **V** and thus close the catalytic cycle. We compared the R-pathway computed with smd solvent model with the same mechanism reevaluated with the pcm solvent model. The comparison is shown in Figure X, energetics vary but show that also with pcm solvation the alternative pathway is less favourable than the pathway presented in the main part of the paper.

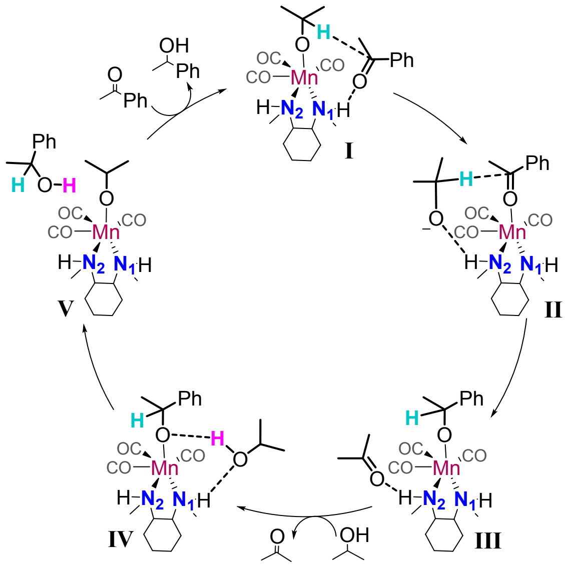

Scheme S1. Alternative proposed Meerwein-Ponndorf-Verley-type mechanism for ketone transfer hydrogenation by the Mn-NN catalyst facilitated by isopropanol solvent molecules

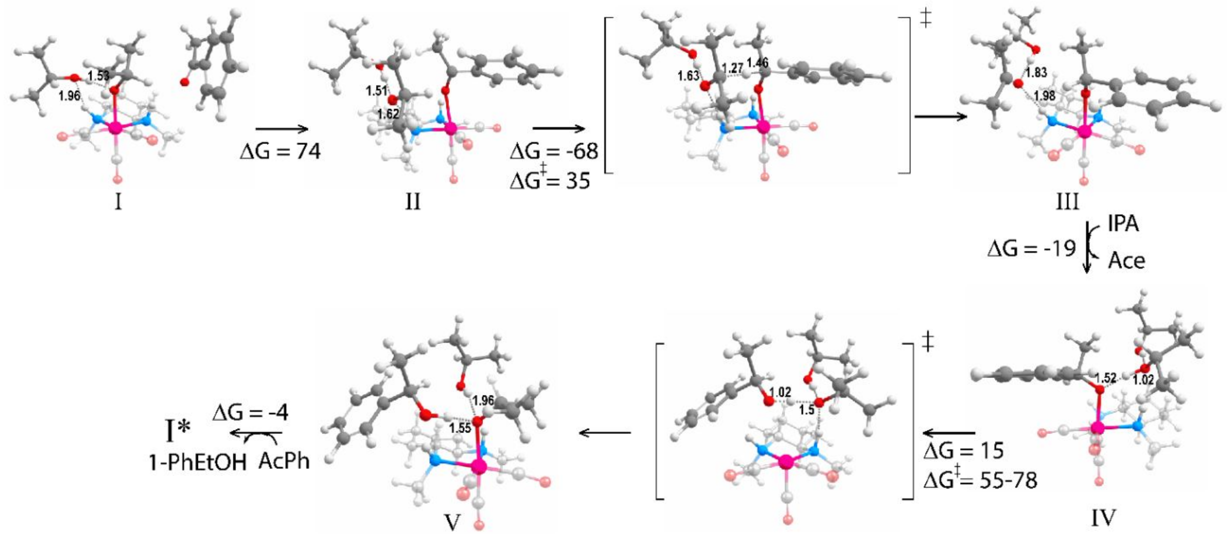

Figure S2. Structures of reaction intermediates and transition states of the alternative MERP for the reduction of acetophenone to R-phenylethanol. Gibbs free energies in kJ mol^{-1}, bond distances in Å.

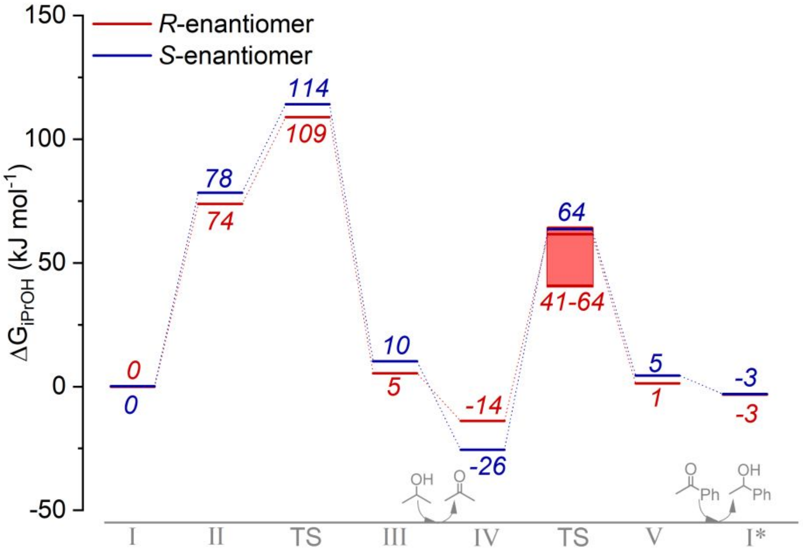

Figure S3. Reaction Gibbs free energy diagram for the reduction of acetophenone to R- and S-1-phenylethanol (red and blue, respectively) in the alternative microsolvation model

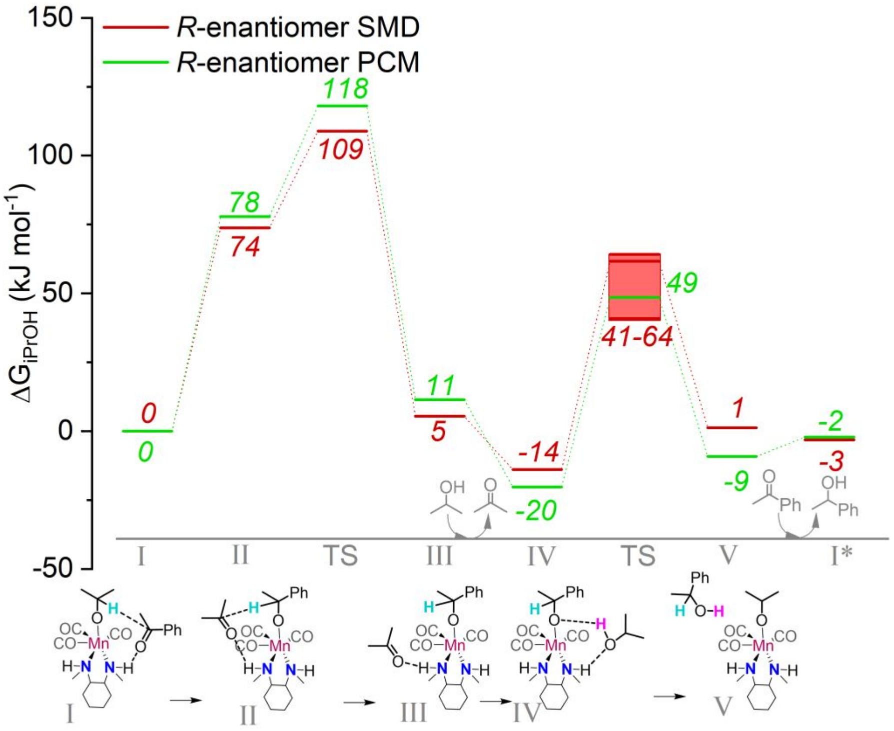

Figure S4. Reaction Gibbs free energy diagram for the reduction of acetophenone to R-phenylethanol in the alternative microsolvation model computed in SMD (red) and PCM (green) implicit solvation model

S4. Energetics of optimized structures of the alternative pathway

Free molecules

Species	E	ZPE	E_{therm}	S
**Acetone**	-192.992428	-192.909018	56.330	74.797
**Isopropanol**	-194.196491	-194.088499	71.865	73.384
**1-Phenylethanol**	-385.764157	-385.602510	107.860	94.175
**Acetophenone**	-384.561701	-384.423543	92.701	91.409

Reaction Intermediates

Species	E	ZPE	E_{therm}	S
**I R**	-2687.785039	-2687.143172	433.442	263.681
**I S**	-2687.785482	-2687.143346	433.520	262.956
**II R**	-2687.755053	-2687.116636	431.076	260.084
**II S**	-2687.754332	-2687.115780	431.074	258.201
**III R**	-2687.778516	-2687.139377	432.113	268.104
**III S**	-2687.777001	-2687.137712	432.182	267.689
**IV R**	-2688.993823	-2688.329348	447.553	259.054
**IV S**	-2688.997822	-2688.333459	447.442	259.574
**V R**	-2688.991019	-2688.325698	447.849	254.353
**V S**	-2688.991389	-2688.323908	449.093	255.065
**II R gas phase**	-2493.515818	-2492.984702	358.610	222.698
**III R gas phase**	-2493.538781	-2493.008026	358.982	232.094
**II R smd**	-2493.536894	-2493.007489	357.549	221.922
**III R smd**	-2493.566098	-2493.036598	357.965	227.039

Transition States

Species	E	ZPE	E_{therm}	S
**$TS_{II\text{-}III\ R}$**	-2687.743514	-2687.106654	429.292	251.315
**$TS_{II\text{-}III\ S}$**	-2687.740185	-2687.103675	429.287	253.812
**$TS_{IV\text{-}V\ R\ SP1}$**	-2688.965866	-2688.305535	443.954	244.908
**$TS_{IV\text{-}V\ R\ SP2}$**	-2688.968174	-2688.306672	445.479	261.908
**$TS_{IV\text{-}V\ R\ SP3}$**	-2688.964357	-2688.301935	445.819	255.145
**$TS_{IV\text{-}V\ S}$**	-2688.963589	-2688.301345	445.710	254.848
**$TS_{II\text{-}III\ R\ gas}$**	-2493.492794	-2492.964342	356.344	217.369
**$TS_{II\text{-}III\ R\ smd}$**	-2493.521638	-2492.993989	355.610	213.131

E: Electronic energy (PBE0-D3/6-311+G(d,p)//SMD) [Hartree]
ZPE: Zero-point Energy [Hartree]
E_{therm}: Internal Thermal Energy [Kcal/Mol] at 333K
S: Entropy from translational degrees of freedom [Cal/Mol-Kelvin] at 333K

Reaction Intermediates PCM model

Species	E	ZPE	E_{therm}	S
**I R**	-2687.77	-2687.13	434.139	264.603
**II R**	-2687.74	-2687.1	431.383	253.573
**III R**	-2687.76	-2687.12	432.876	270.552
**IV R**	-2688.98	-2688.31	448.33	259.448
**V R**	-2688.98	-2688.31	448.682	255.747

Transition States PCM model

Species	E	ZPE	E_{therm}	S
**$TS_{II\text{-}III\ R}$**	-2687.73	-2687.09	430.208	248.136
**$TS_{IV\text{-}V\ R}$**	-2688.95	-2688.29	446.698	256.578

E: Electronic energy (PBE0-D3/6-311+G(d,p)//PCM) [Hartree]
ZPE: Zero-point Energy [Hartree]
E_{therm}: Internal Thermal Energy [Kcal/Mol] at 333K
S: Entropy from translational degrees of freedom [Cal/Mol-Kelvin] at 333K
